# Crystal structure of cyprodinil

**DOI:** 10.1107/S2056989014025742

**Published:** 2015-01-01

**Authors:** Youngeun Jeon, Gihaeng Kang, Seonghwa Cho, Tae Ho Kim

**Affiliations:** a Department of Chemistry and Research Institute of Natural Sciences, Gyeongsang National University, Jinju 660-701, Republic of Korea

**Keywords:** crystal structure, cyprodinil, pyrimidin-2-amine, fungicide, hydrogen bonding, π–π inter­actions

## Abstract

In the title compound, C_14_H_15_N_3_ (systematic name: 4-cyclo­propyl-6-methyl-*N*-phenyl­pyrimidin-2-amine), which is the anilino­pyrimidine fungicide cyprodinil, the dihedral angles between the planes of the central pyrimidine ring and the terminal phenyl ring and the mean plane of the cyclo­propane ring system are 14.52 (11) and 88.79 (10)°, respectively. In the crystal, weak π–π inter­actions [3.8551 (11) Å] connect the dimers into chains along the *b*-axis direction.

## Related literature   

For information on the fungicidal properties of the title compound, see: Sapp *et al.* (2003 ▸). For a related crystal structure, see: Kang *et al.* (2014 ▸).
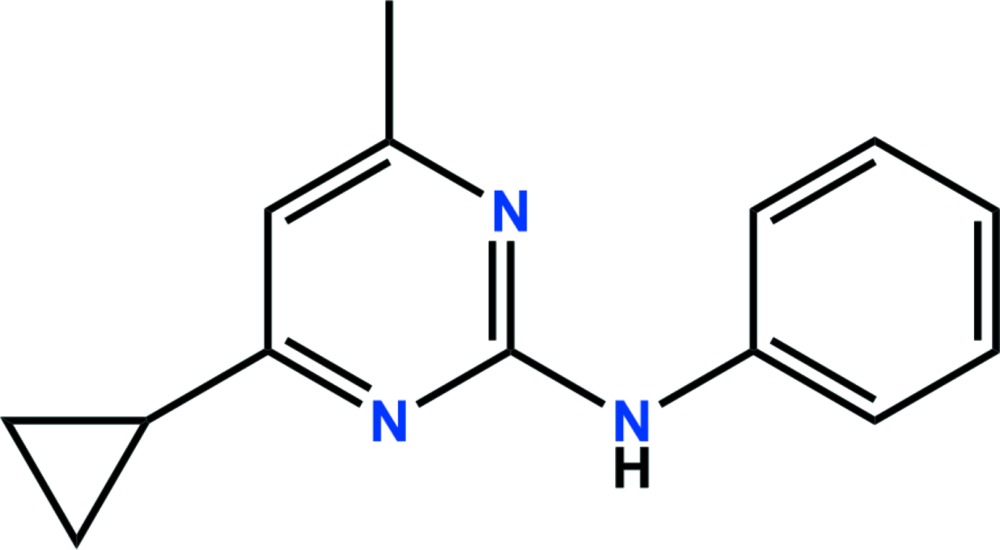


## Experimental   

### Crystal data  

C_14_H_15_N_3_
*M* *_r_* = 225.29
Monoclinic, 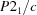

*a* = 13.1920 (6) Å
*b* = 5.3176 (2) Å
*c* = 16.8641 (7) Å
β = 100.288 (2)°
*V* = 1163.99 (8) Å^3^
*Z* = 4
Mo *K*α radiation
μ = 0.08 mm^−1^
*T* = 173 K
0.45 × 0.22 × 0.18 mm

### Data collection  

Bruker APEXII CCD diffractometer
Absorption correction: multi-scan (*SADABS*; Bruker, 2009 ▸) *T*_min_ = 0.965, *T*_max_ = 0.986
18116 measured reflections
2864 independent reflections
2463 reflections with *I* > 2σ(*I*)
*R*_int_ = 0.056

### Refinement  

*R*[*F* ^2^ > 2σ(*F* ^2^)] = 0.056
*wR*(*F* ^2^) = 0.166
*S* = 1.14
2864 reflections
155 parameters
H-atom parameters constrained
Δρ_max_ = 0.29 e Å^−3^
Δρ_min_ = −0.31 e Å^−3^

### 

Data collection: *APEX2* (Bruker, 2009 ▸); cell refinement: *SAINT* (Bruker, 2009 ▸); data reduction: *SAINT*; program(s) used to solve structure: *SHELXTL* (Sheldrick, 2008 ▸); program(s) used to refine structure: *SHELXTL*; molecular graphics: *DIAMOND* (Brandenburg, 2010 ▸); software used to prepare material for publication: *SHELXTL*.

## Supplementary Material

Crystal structure: contains datablock(s) global, I. DOI: 10.1107/S2056989014025742/hg5422sup1.cif

Structure factors: contains datablock(s) I. DOI: 10.1107/S2056989014025742/hg5422Isup2.hkl

Supporting information file. DOI: 10.1107/S2056989014025742/hg5422Isup3.cml

**Fig. 1.**
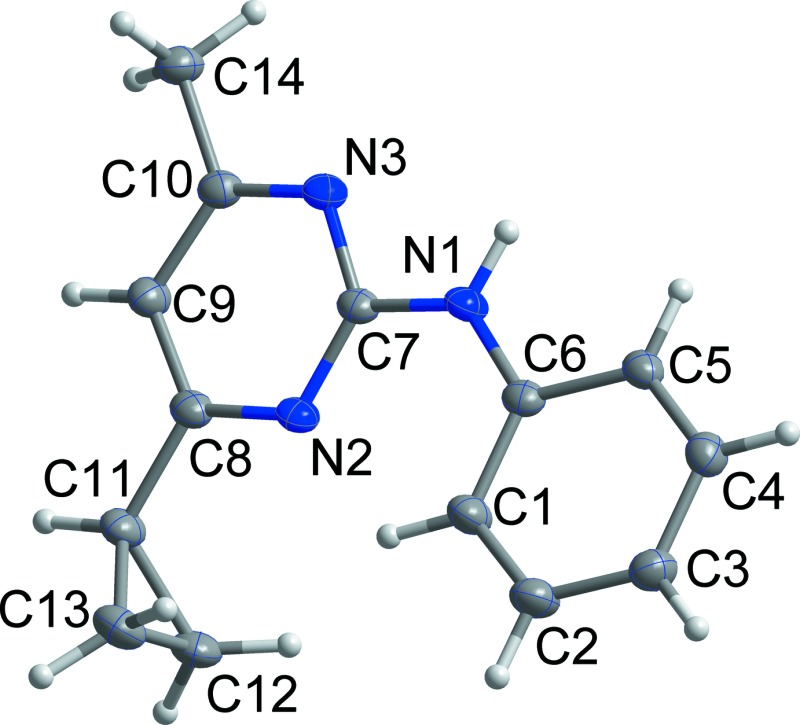
The asymmetric unit of the title compound with the atom numbering scheme. Displacement ellipsoids are drawn at the 50% probability level. H atoms are shown as small spheres of arbitrary radius.

**Fig. 2.**
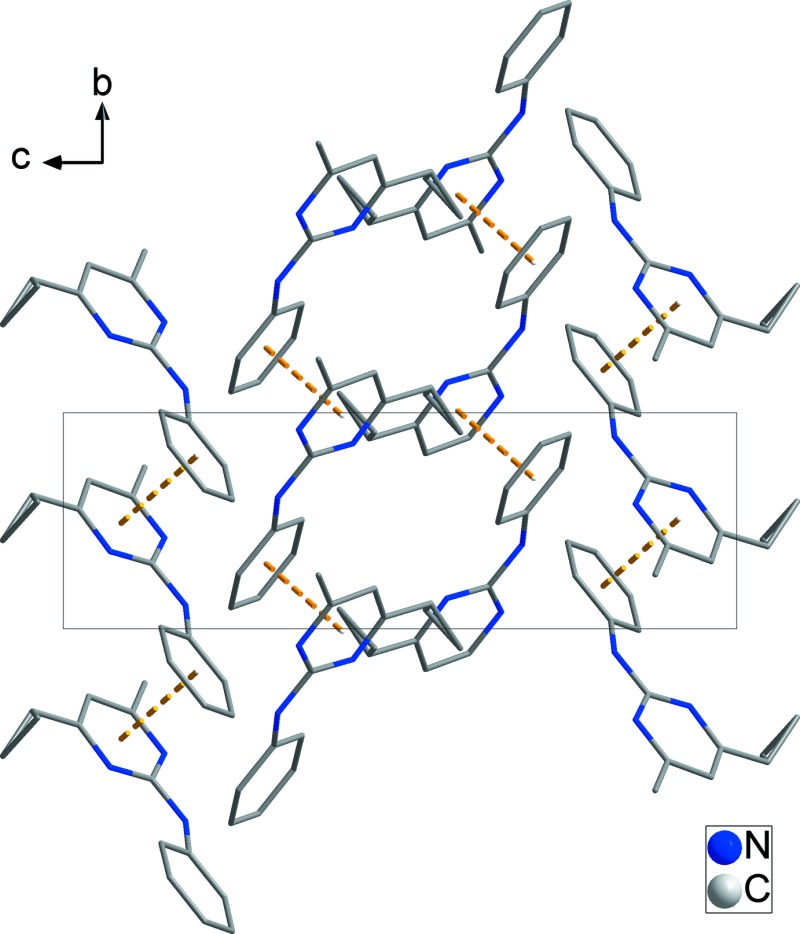
Crystal packing viewed along the a axis. The weak π–π interactions are shown as dashed lines.

. DOI: 10.1107/S2056989014025742/hg5422fig1.tif
The asymmetric unit of the title compound with the atom numbering scheme. Displacement ellipsoids are drawn at the 50% probability level. H atoms are shown as small spheres of arbitrary radius.

a . DOI: 10.1107/S2056989014025742/hg5422fig2.tif
Crystal packing viewed along the *a* axis. The inter­molecular N—H⋯N hydrogen bonds, and weak π–π inter­actions are shown as dashed lines.

CCDC reference: 1035821

Additional supporting information:  crystallographic information; 3D view; checkCIF report

## supplementary crystallographic information

### S1. Comment

Cyprodinil, C_14_H_15_N_3_, is a systemic pyrimidine fungicide for foliar applications on cereals and strawberries against plant pathogenic fungi (Sapp *et al.*, 2003). Its crystal structure is reported herein. In this compound (Scheme 1, Fig. 1), the dihedral angles between the central pyrimidine ring and the terminal phenyl ring and mean plane of cyclopropane ring system are 14.52 (11) and 88.79 (10)°. All bond lengths and bond angles are normal and comparable to those observed in the crystal structure of a similar compound (Kang *et al.*, 2014).

In the crystal structure (Fig. 2), The crystal structure is stabilized by weak intermolecular π–π interaction between the pyrimidine ring and terminal phenyl ring systems [*C*g1···*C*g2^ii^, 3.8552 (11) Å] are present (*C*g1 and *C*g2 are the centroids of the C1—C6 and C7—N2—C8—C9—C10—N3 rings, respectively) [for symmetry codes: (ii), *x*, *y* - 1, *z*].

### S2. Experimental

The title compound was purchased from the Chem Servies Company. Slow evaporation of a solution in CH_2_Cl_2_ gave single crystals suitable for X-ray analysis.

### S3. Refinement

All H-atoms were positioned geometrically and refined using a riding model with d(N—H) = 0.88 Å, *U*_iso_ = 1.2*U*_eq_(C) for amine group, d(C—H) = 0.98 Å, *U*_iso_ = 1.5*U*_eq_(C) for methyl group, d(C—H) = 0.99 Å, *U*_iso_ = 1.2*U*_eq_(C) for C*sp*^3^—H, d(C—H) = 1.00 Å, *U*_iso_ = 1.2*U*_eq_(C) for C*sp*^3^—H, and d(C—H) = 0.95 Å, *U*_iso_ = 1.2*U*_eq_(C) for aromatic C—H.

### Crystal data

**Table:**

C_14_H_15_N_3_	*F*(000) = 480
*M**_r_* = 225.29	*D*_x_ = 1.286 Mg m^−^^3^
Monoclinic, *P*2_1_/*c*	Mo *K*α radiation, λ = 0.71073 Å
Hall symbol: -P 2ybc	Cell parameters from 8168 reflections
*a* = 13.1920 (6) Å	θ = 2.5–28.2°
*b* = 5.3176 (2) Å	µ = 0.08 mm^−^^1^
*c* = 16.8641 (7) Å	*T* = 173 K
β = 100.288 (2)°	Block, colourless
*V* = 1163.99 (8) Å^3^	0.45 × 0.22 × 0.18 mm
*Z* = 4	

### Data collection

**Table:**

Bruker APEXII CCD diffractometer	2864 independent reflections
Radiation source: fine-focus sealed tube	2463 reflections with *I* > 2σ(*I*)
Graphite monochromator	*R*_int_ = 0.056
φ and ω scans	θ_max_ = 28.3°, θ_min_ = 1.6°
Absorption correction: multi-scan (*SADABS*; Bruker, 2009)	*h* = −17→17
*T*_min_ = 0.965, *T*_max_ = 0.986	*k* = −7→7
18116 measured reflections	*l* = −21→22

### Refinement

**Table:**

Refinement on *F*^2^	Primary atom site location: structure-invariant direct methods
Least-squares matrix: full	Secondary atom site location: difference Fourier map
*R*[*F*^2^ > 2σ(*F*^2^)] = 0.056	Hydrogen site location: inferred from neighbouring sites
*wR*(*F*^2^) = 0.166	H-atom parameters constrained
*S* = 1.14	*w* = 1/[σ^2^(*F*_o_^2^) + (0.0577*P*)^2^ + 1.3715*P*] where *P* = (*F*_o_^2^ + 2*F*_c_^2^)/3
2864 reflections	(Δ/σ)_max_ < 0.001
155 parameters	Δρ_max_ = 0.29 e Å^−^^3^
0 restraints	Δρ_min_ = −0.31 e Å^−^^3^

### Special details

**Table:**

Geometry. All e.s.d.'s (except the e.s.d. in the dihedral angle between two l.s. planes) are estimated using the full covariance matrix. The cell e.s.d.'s are taken into account individually in the estimation of e.s.d.'s in distances, angles and torsion angles; correlations between e.s.d.'s in cell parameters are only used when they are defined by crystal symmetry. An approximate (isotropic) treatment of cell e.s.d.'s is used for estimating e.s.d.'s involving l.s. planes.
Refinement. Refinement of *F*^2^ against ALL reflections. The weighted *R*-factor *wR* and goodness of fit *S* are based on *F*^2^, conventional *R*-factors *R* are based on *F*, with *F* set to zero for negative *F*^2^. The threshold expression of *F*^2^ > σ(*F*^2^) is used only for calculating *R*-factors(gt) *etc*. and is not relevant to the choice of reflections for refinement. *R*-factors based on *F*^2^ are statistically about twice as large as those based on *F*, and *R*- factors based on ALL data will be even larger.

### Fractional atomic coordinates and isotropic or equivalent isotropic displacement parameters (Å^2^)

**Table:**

	*x*	*y*	*z*	*U*_iso_*/*U*_eq_	
N1	0.64381 (12)	0.1086 (3)	0.81833 (9)	0.0210 (3)	
H1N	0.5882	0.0697	0.7834	0.025*	
N2	0.70730 (12)	0.3584 (3)	0.93020 (9)	0.0201 (3)	
N3	0.53921 (12)	0.4215 (3)	0.84955 (9)	0.0206 (3)	
C1	0.83033 (14)	0.0059 (4)	0.85440 (11)	0.0246 (4)	
H1	0.8436	0.1437	0.8906	0.030*	
C2	0.90995 (15)	−0.1513 (4)	0.84207 (12)	0.0277 (4)	
H2	0.9778	−0.1183	0.8700	0.033*	
C3	0.89324 (15)	−0.3555 (4)	0.79011 (12)	0.0256 (4)	
H3	0.9486	−0.4621	0.7827	0.031*	
C4	0.79368 (15)	−0.4010 (4)	0.74907 (11)	0.0241 (4)	
H4	0.7808	−0.5396	0.7131	0.029*	
C5	0.71331 (14)	−0.2457 (4)	0.76022 (11)	0.0208 (4)	
H5	0.6458	−0.2787	0.7318	0.025*	
C6	0.73029 (14)	−0.0402 (4)	0.81301 (10)	0.0195 (4)	
C7	0.63140 (14)	0.3035 (4)	0.86853 (10)	0.0189 (4)	
C8	0.69016 (14)	0.5539 (4)	0.97648 (10)	0.0208 (4)	
C9	0.59859 (15)	0.6871 (4)	0.96202 (11)	0.0233 (4)	
H9	0.5873	0.8251	0.9951	0.028*	
C10	0.52353 (14)	0.6127 (4)	0.89742 (11)	0.0209 (4)	
C11	0.77222 (15)	0.6184 (4)	1.04550 (11)	0.0246 (4)	
H11	0.7567	0.7622	1.0796	0.029*	
C12	0.83749 (15)	0.4101 (4)	1.08962 (12)	0.0276 (4)	
H12A	0.8585	0.4258	1.1488	0.033*	
H12B	0.8239	0.2360	1.0698	0.033*	
C13	0.88371 (16)	0.5880 (4)	1.03804 (12)	0.0300 (5)	
H13A	0.8989	0.5239	0.9863	0.036*	
H13B	0.9334	0.7136	1.0653	0.036*	
C14	0.42255 (15)	0.7483 (4)	0.87894 (12)	0.0255 (4)	
H14A	0.4283	0.8895	0.8427	0.038*	
H14B	0.4043	0.8118	0.9291	0.038*	
H14C	0.3690	0.6324	0.8530	0.038*	

### Atomic displacement parameters (Å^2^)

**Table:**

	*U*^11^	*U*^22^	*U*^33^	*U*^12^	*U*^13^	*U*^23^
N1	0.0185 (7)	0.0259 (8)	0.0165 (7)	0.0004 (6)	−0.0023 (5)	−0.0027 (6)
N2	0.0205 (7)	0.0238 (8)	0.0145 (7)	0.0001 (6)	−0.0009 (5)	0.0008 (6)
N3	0.0196 (7)	0.0250 (8)	0.0164 (7)	0.0014 (6)	0.0010 (5)	0.0005 (6)
C1	0.0218 (9)	0.0278 (10)	0.0221 (9)	−0.0006 (8)	−0.0016 (7)	−0.0040 (8)
C2	0.0209 (9)	0.0329 (11)	0.0270 (9)	0.0014 (8)	−0.0016 (7)	−0.0027 (8)
C3	0.0242 (9)	0.0279 (10)	0.0243 (9)	0.0047 (8)	0.0031 (7)	0.0001 (8)
C4	0.0290 (10)	0.0234 (10)	0.0194 (8)	−0.0003 (8)	0.0027 (7)	−0.0025 (7)
C5	0.0226 (9)	0.0220 (9)	0.0166 (8)	−0.0016 (7)	0.0002 (6)	0.0006 (7)
C6	0.0207 (8)	0.0227 (9)	0.0146 (7)	0.0004 (7)	0.0014 (6)	0.0019 (7)
C7	0.0200 (8)	0.0221 (9)	0.0138 (7)	0.0002 (7)	0.0007 (6)	0.0016 (7)
C8	0.0227 (9)	0.0233 (9)	0.0151 (8)	−0.0009 (7)	0.0005 (6)	0.0016 (7)
C9	0.0258 (9)	0.0246 (10)	0.0181 (8)	0.0028 (8)	0.0001 (7)	−0.0033 (7)
C10	0.0219 (9)	0.0235 (9)	0.0167 (8)	0.0015 (7)	0.0018 (6)	0.0022 (7)
C11	0.0250 (9)	0.0284 (10)	0.0180 (8)	0.0005 (8)	−0.0025 (7)	−0.0035 (7)
C12	0.0244 (9)	0.0333 (11)	0.0216 (9)	0.0006 (8)	−0.0053 (7)	0.0005 (8)
C13	0.0245 (9)	0.0382 (12)	0.0247 (9)	−0.0057 (8)	−0.0022 (7)	−0.0022 (9)
C14	0.0232 (9)	0.0289 (10)	0.0226 (9)	0.0054 (8)	−0.0005 (7)	−0.0004 (8)

### Geometric parameters (Å, º)

**Table:**

N1—C7	1.367 (2)	C5—H5	0.9500
N1—C6	1.404 (2)	C8—C9	1.384 (3)
N1—H1N	0.8800	C8—C11	1.481 (2)
N2—C7	1.341 (2)	C9—C10	1.392 (3)
N2—C8	1.343 (2)	C9—H9	0.9500
N3—C10	1.337 (2)	C10—C14	1.498 (3)
N3—C7	1.355 (2)	C11—C13	1.507 (3)
C1—C2	1.387 (3)	C11—C12	1.514 (3)
C1—C6	1.400 (2)	C11—H11	1.0000
C1—H1	0.9500	C12—C13	1.488 (3)
C2—C3	1.388 (3)	C12—H12A	0.9900
C2—H2	0.9500	C12—H12B	0.9900
C3—C4	1.392 (3)	C13—H13A	0.9900
C3—H3	0.9500	C13—H13B	0.9900
C4—C5	1.383 (3)	C14—H14A	0.9800
C4—H4	0.9500	C14—H14B	0.9800
C5—C6	1.402 (3)	C14—H14C	0.9800
			
C7—N1—C6	130.97 (15)	C8—C9—H9	121.0
C7—N1—H1N	114.5	C10—C9—H9	121.0
C6—N1—H1N	114.5	N3—C10—C9	121.56 (17)
C7—N2—C8	116.06 (16)	N3—C10—C14	117.93 (16)
C10—N3—C7	116.01 (16)	C9—C10—C14	120.50 (17)
C2—C1—C6	119.45 (18)	C8—C11—C13	119.78 (16)
C2—C1—H1	120.3	C8—C11—C12	119.22 (18)
C6—C1—H1	120.3	C13—C11—C12	58.99 (14)
C1—C2—C3	121.81 (18)	C8—C11—H11	115.7
C1—C2—H2	119.1	C13—C11—H11	115.7
C3—C2—H2	119.1	C12—C11—H11	115.7
C2—C3—C4	118.62 (18)	C13—C12—C11	60.26 (14)
C2—C3—H3	120.7	C13—C12—H12A	117.7
C4—C3—H3	120.7	C11—C12—H12A	117.7
C5—C4—C3	120.47 (18)	C13—C12—H12B	117.7
C5—C4—H4	119.8	C11—C12—H12B	117.7
C3—C4—H4	119.8	H12A—C12—H12B	114.9
C4—C5—C6	120.82 (17)	C12—C13—C11	60.74 (14)
C4—C5—H5	119.6	C12—C13—H13A	117.7
C6—C5—H5	119.6	C11—C13—H13A	117.7
C1—C6—C5	118.84 (17)	C12—C13—H13B	117.7
C1—C6—N1	125.02 (17)	C11—C13—H13B	117.7
C5—C6—N1	116.12 (16)	H13A—C13—H13B	114.8
N2—C7—N3	126.65 (17)	C10—C14—H14A	109.5
N2—C7—N1	119.35 (16)	C10—C14—H14B	109.5
N3—C7—N1	114.00 (16)	H14A—C14—H14B	109.5
N2—C8—C9	121.73 (17)	C10—C14—H14C	109.5
N2—C8—C11	117.43 (17)	H14A—C14—H14C	109.5
C9—C8—C11	120.82 (17)	H14B—C14—H14C	109.5
C8—C9—C10	117.96 (18)		
			
C6—C1—C2—C3	−0.5 (3)	C6—N1—C7—N3	172.68 (17)
C1—C2—C3—C4	0.5 (3)	C7—N2—C8—C9	1.2 (3)
C2—C3—C4—C5	−0.2 (3)	C7—N2—C8—C11	179.58 (16)
C3—C4—C5—C6	−0.1 (3)	N2—C8—C9—C10	0.0 (3)
C2—C1—C6—C5	0.2 (3)	C11—C8—C9—C10	−178.32 (18)
C2—C1—C6—N1	−178.05 (18)	C7—N3—C10—C9	1.0 (3)
C4—C5—C6—C1	0.1 (3)	C7—N3—C10—C14	−179.81 (17)
C4—C5—C6—N1	178.52 (17)	C8—C9—C10—N3	−1.2 (3)
C7—N1—C6—C1	−8.8 (3)	C8—C9—C10—C14	179.64 (18)
C7—N1—C6—C5	172.85 (18)	N2—C8—C11—C13	35.3 (3)
C8—N2—C7—N3	−1.5 (3)	C9—C8—C11—C13	−146.3 (2)
C8—N2—C7—N1	179.34 (16)	N2—C8—C11—C12	−33.6 (3)
C10—N3—C7—N2	0.4 (3)	C9—C8—C11—C12	144.84 (19)
C10—N3—C7—N1	179.63 (16)	C8—C11—C12—C13	109.1 (2)
C6—N1—C7—N2	−8.0 (3)	C8—C11—C13—C12	−108.2 (2)
