# Supplementary figures and images for: Crystal structure of cyprodinil
Source: Acta Crystallogr E Crystallogr Commun. 2015 Jan 1;71(Pt 1):o5. doi: 10.1107/S2056989014025742 (PMC4331914; doi:10.1107/S2056989014025742)

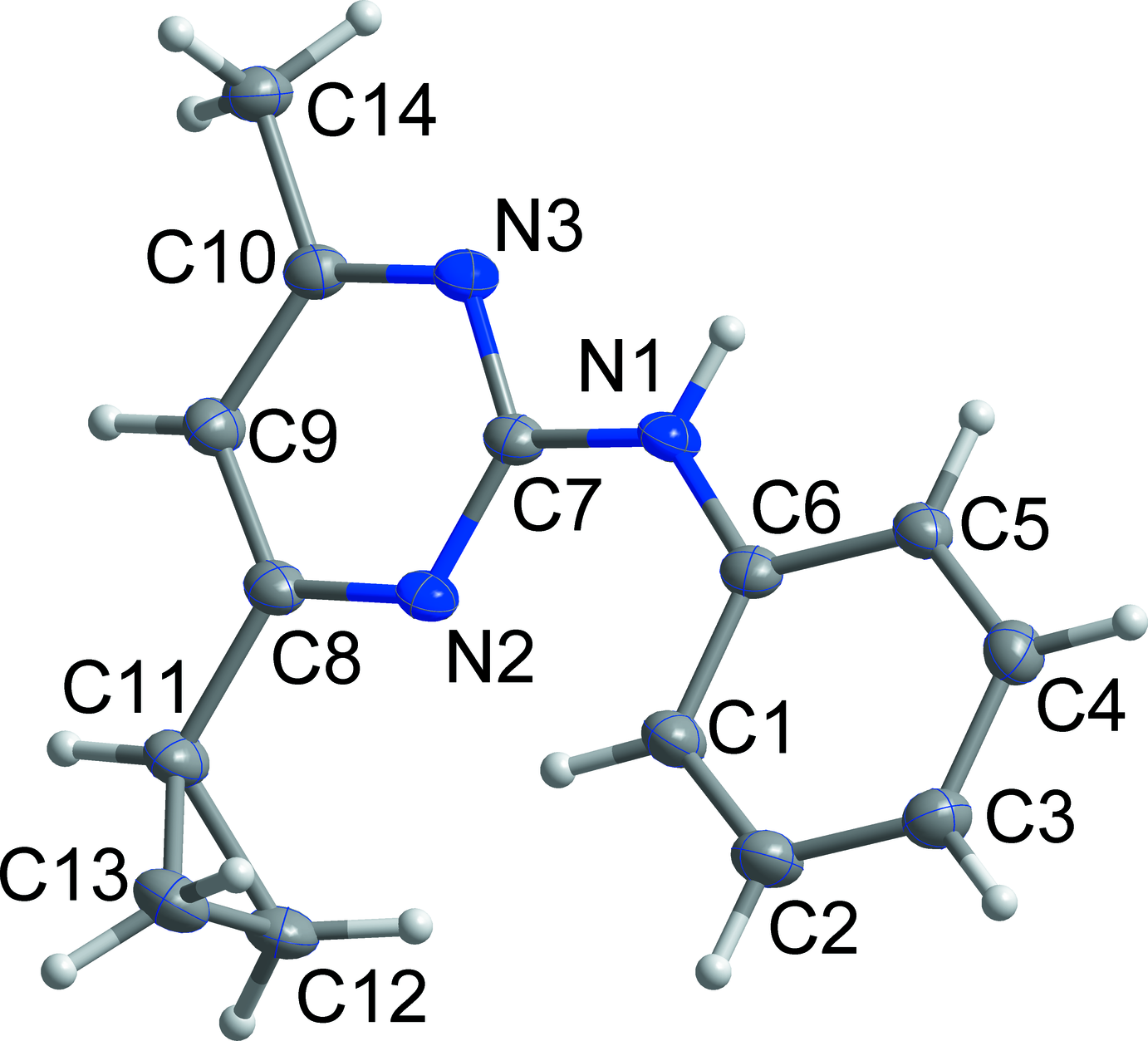

Supplement: Supplementary file 4 [file e-71-000o5-fig1.tif]

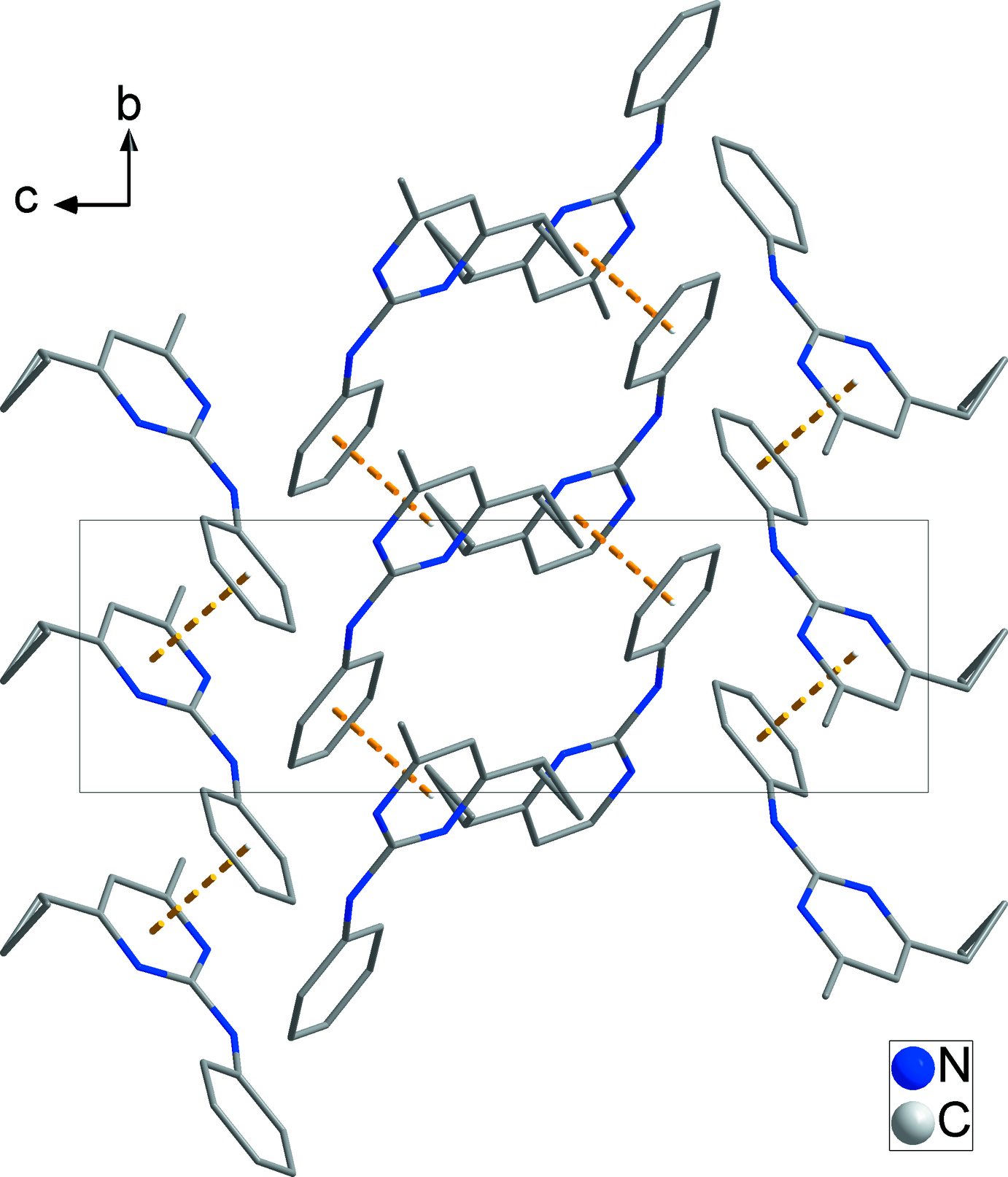

Supplement: Supplementary file 5 [file e-71-000o5-fig2.tif]
